# Influence of Cr_3_C_2_ and VC Content on WC Grain Size, WC Shape and Mechanical Properties of WC–6.0 wt. % Co Cemented Carbides

**DOI:** 10.3390/ma14061551

**Published:** 2021-03-22

**Authors:** Chao Yin, Yingbiao Peng, Jianming Ruan, Lin Zhao, Ren Zhang, Yong Du

**Affiliations:** 1 State Key Laboratory for Powder Metallurgy, Central South University, Changsha 410083, China; yinchaozb@126.com (C.Y.); jianming@csu.edu.cn (J.R.); yong-du@csu.edu.cn (Y.D.); 2 State Key Laboratory of Cemented Carbide, Zhuzhou Cemented Carbide Group Co., LTD., Zhuzhou 412000, China; zhaolin@csu.edu.cn (L.Z.); zhangren1988@163.com (R.Z.); 3 College of Metallurgy and Material Engineering, Hunan University of Technology, Zhuzhou 412008, China

**Keywords:** Cr_3_C_2_, VC, cemented carbide, grain size, WC shape, mechanical properties

## Abstract

In this paper, the influences of Cr_3_C_2_/VC content on WC grain size, WC grain shape and mechanical properties of WC–6 wt. % Co cemented carbides were investigated. The results showed that the grain size first rapidly decreased and then slightly decreased with the increasing Cr_3_C_2_/VC content, and VC led to finer grain size and narrower size distribution. HRTEM/EDS analysis of the WC/Co interface indicates that the segregation concentration of V is much larger than that of Cr, which may be a strong response to the higher inhibition efficiency of VC. The addition of Cr_3_C_2_ induced triangular prism shape WC grains while VC generated stepped triangular prism grains. Despite the grain growth inhibitor (GGI) mechanisms of Cr_3_C_2_/VC have been extensively studied in the literature, the doping amount, especially the doping limit, has not been systematically investigated. In this work, the saturated solubilities of Cr and V in cobalt binder phase along with carbon content hare been predicted based on thermodynamic calculations. Based on the theoretical calculations, the doping amount of Cr_3_C_2_/VC is designed to be gradually increasing until more or less over their maximum solubilities in the binder phase, thereby investigating the subsequent microstructure and mechanical properties. When the doping of Cr_3_C_2_/VC exceeds the maximum solubility in Co phase, Co-rich Cr-carbides and Co-deficient V-carbides would form respectively, which were detrimental to the transverse rupture strength (TRS) and impact toughness. The hardness increased with the increasing Cr_3_C_2_/VC content, while the fracture toughness decreased with the increasing Cr_3_C_2_/VC content. The TRS initially enhanced and then declined, but the stepped triangular prism shape grains and low fraction of WC/Co interface in WC–6Co–VC cemented carbide led to a more pronounced decline in the TRS. The sample with 0.6 wt. % Cr_3_C_2_ addition had good comprehensive mechanical properties, its hardness, fracture toughness and TRS were 1880 kg/mm^2^, 9.32 MPa·m^1/2^ and 3450 MPa, respectively.

## 1. Introduction

Compared to micron-grained WC-Co cemented carbides, ultrafine/nano-grained cemented carbides exhibit better mechanical properties, such as hardness, fracture toughness, transverse rupture strength, and so on [1,2,3]. However, the commonly used method for fabricating ultrafine/nano–grained cemented carbides mostly required ultrafine or nano size initial powder, which enhances the surface reactivity and increase the possibility of abnormal grain growth according to Ostwald-ripening mechanism [4]. Thus, the key technique for preparing ultrafine/nano–grained cemented carbides is to control the growth of WC grains. For this purpose, plenty of efforts have been paid to retard abnormal growth of WC grains [5,6,7]. Till now, the most successful method of controlling the WC grain growth is the addition of small amounts (less than 1 wt. %) of grain growth inhibitors (GGI), such as VC, Cr_3_C_2_, TaC, NbC, TiC or Mo_2_C [8,9,10,11]. Among various inhibitors, VC and Cr_3_C_2_ are the most effective GGI [12].

In the last few decades, Cr_3_C_2_ and VC as GGI have been studied by many researchers. Studies have shown that with the addition of Cr_3_C_2_ and VC, Cr and V are prone to segregate at the WC/Co grain boundaries as (Cr, W) C_x_ and (V, W) C_x_ segregation layers [13,14,15,16,17], thus suppressing the WC grain growth during sintering. Commonly, the inhibition efficiency increases with rising Cr and V concentration. Meanwhile, Cr_3_C_2_ and VC can reduce the defect (such as pore) size and improve microstructure uniformity [18]. Therefore, the bonding strength and total amount of interfacial area between the WC grains and the Co binder is substantially increased, resulting in a remarkable improvement in mechanical properties. Moreover, Cr_3_C_2_ and VC also play an important role in WC grain morphology. VC induces sharp triangular grains with multi–steps while Cr_3_C_2_ generates partly rounded WC grains [9,19,20,21]. However, the superfluous addition of Cr_3_C_2_ and VC can reduce WC/Co interface coherency, and low fraction of WC/Co interfacial coherency is detrimental to the TRS [13,14]. Furthermore, when the GGI content exceeds the maximum solubility in the binder phase, GGI–based carbides precipitate will form, which has negative consequences on mechanical properties of cemented carbides [2,6,22,23]. Therefore, the amount of grain growth inhibitors added should be precisely controlled, which is crucial to the microstructure and mechanical properties of cemented carbides.

In order to further optimize the mechanical properties of ultrafine cemented carbides with the addition of Cr_3_C_2_ and VC, it is necessary to investigate the saturated solubilities of GGIs in cobalt binder phase as well as the effect of GGIs, doping more or less than their maximum solubilities, on WC grain size, WC morphology and mechanical properties of ultra-fine cemented carbides. In the present work, the saturated solubilities of Cr_3_C_2_ and VC were predicted based on thermodynamic calculations, which were further utilized to analyze the influence of Cr_3_C_2_/VC content on mechanical properties of WC–6Co–(Cr_3_C_2_/VC) cemented carbides. The present work may provide a strategy to optimize the microstructures and achieve high comprehensive properties of the ultrafine cemented carbides.

## 2. Experimental

### 2.1. Materials Preparation

**Figure 1:**
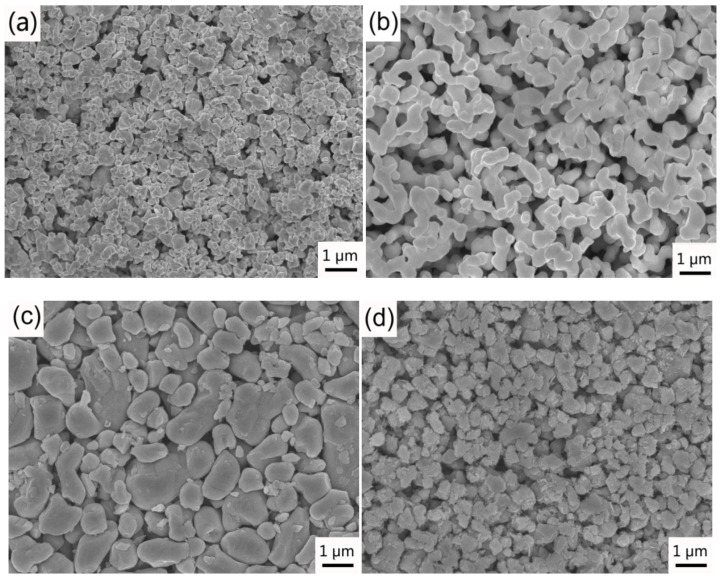
SEM images of (**a**) WC; (**b**) Co; (**c**) Cr_3_C_2_ and (**d**) VC raw powders.

WC powder (purity: 99.95%, 0.6 μm average size), spherical Co powder (purity: 99.94%, 0.6 μm average size), Cr_3_C_2_ powder (purity: 99.96%, 1.2 μm average size) and VC powder (purity: 99.95%, 0.8 μm average size) supplied by Zhuzhou Cemented Carbide Group Corp. Ltd. (Zhuzhou, China) were used as raw materials. SEM images of raw powders were shown in Figure 1. The compositions of the WC–6Co–(Cr_2_C_3_/VC) cemented carbides prepared in the present work are listed in Table 1. All samples were prepared by conventional powder metallurgical technology. The powder mixtures containing 2.0 wt. % paraffin wax were milled at the ball-to-powder ratio of 6:1 for 40 h using ethanol as a medium. The powder mixture was then dried under vacuum at 85 °C and pressed under a uniaxial pressure of 150 MPa. These samples were sintered in the Ar atmosphere with 5 MPa pressure at 1450 °C for 1 h, and cooled with furnace. The samples with the dimension of 5.25 × 6.5 × 20 mm^3^ were prepared for microstructure observation and mechanical properties test.

### 2.2. Characterization

The sintered samples were ground and polished by diamond pastes. The phases in the sintered samples were analyzed by X-ray diffraction (XRD, D8 Advance, Bruker, Karlsrush, Germany) with copper Kα radiation. The microstructure of sintered samples was observed by scanning electron microscopy (SEM, JSM-6701F, JEOL, Tokyo, Japan) equipped with energy dispersive spectroscopy (EDS, AZtec Energy Standard X–MaxN80X, JEOL, Tokyo, Japan). WC grains in the sintered samples were extracted by placing samples in a saturation hydrochloric and FeCl_3_ solution for 40 min to remove Co binder matrix, and then was observed by SEM. The grain size was measured via linear–intercept method based on SEM images. Focused ion beam (FIB) in a SEM (Helios 600i NanoLab, FEI, Oregon, OR, USA) was applied to the preparation of TEM specimens. High resolution transmission electron microscope (HRTEM, Titan G2 60-300, FEI, Oregon, OR, USA) was used to detect the WC/Co interface by doping Cr_3_C_2_ and VC, as well as the elemental mapping of Cr and V through the equipped EDX detector.

Hardness was measured by Vicker’s hardness tester (FV–700, Future Tech, Tokyo, Japan) under a constant load of 30 kg for a dwell time of 15 s, and the fracture toughness (*K*_IC_) value was calculated by the Palmqvist equation [24] based on the crack length at four corners of the indentation. The TRS was measured by three–point transverse method on a universal material testing machine (Instron3369, Instron, Norwood, MA, USA). Six individual TRS and hardness were performed for each sample to generate an average value.

## 3. Results and Discussion

### 3.1. Microstructure and Phase Constitution Analysis

**Figure 2:**
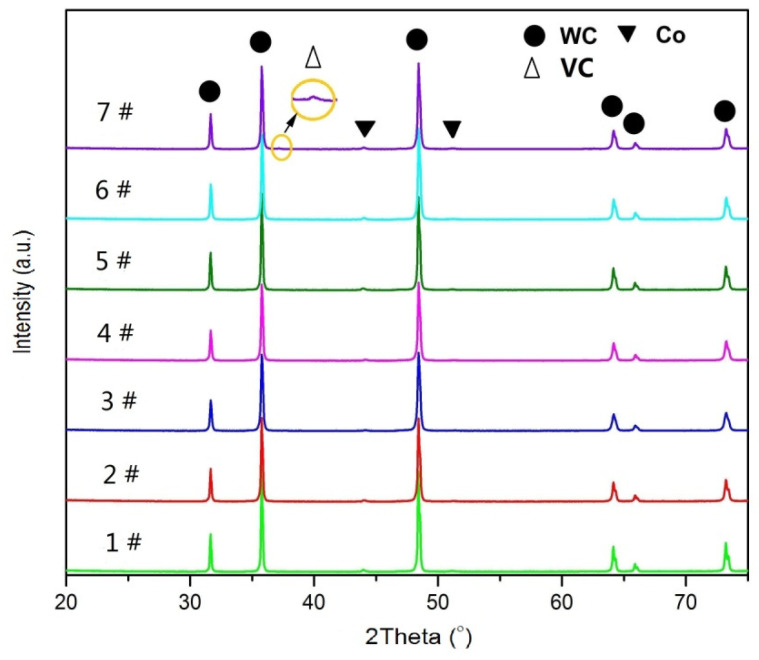
XRD patterns of WC–6Co–(Cr_2_C_3_/VC) cemented carbides sintered at 1450 °C. 1#: WC–6Co; 2#: WC–6Co–0.25Cr_3_C_2_; 3#: WC–6Co–0.6Cr_3_C_2_; 4#: WC–6Co–1.0Cr_3_C_2_; 5#: WC–6Co–0.25VC; 6#: WC–6Co–0.6VC; 7#: WC–6Co–1.0VC.

Figure 2 shows the XRD patterns of the WC-6Co-(Cr_2_C_3_/VC) samples sintered at 1450 °C for 1 h. It is clear that no graphite or η phases are formed in all samples and only WC and Co phases can be observed in samples 1#–6#. This result is consistent with the reports by Chen et al. and Wang et al. [15,16]. However, the diffraction peaks of VC is detected in the XRD patterns as 1.0 wt. % VC is added (sample 7#). The explanation for this phenomenon could be that the addition of VC exceeds its maximum solubility in Co phase and VC precipitate forms.

**Figure 3:**
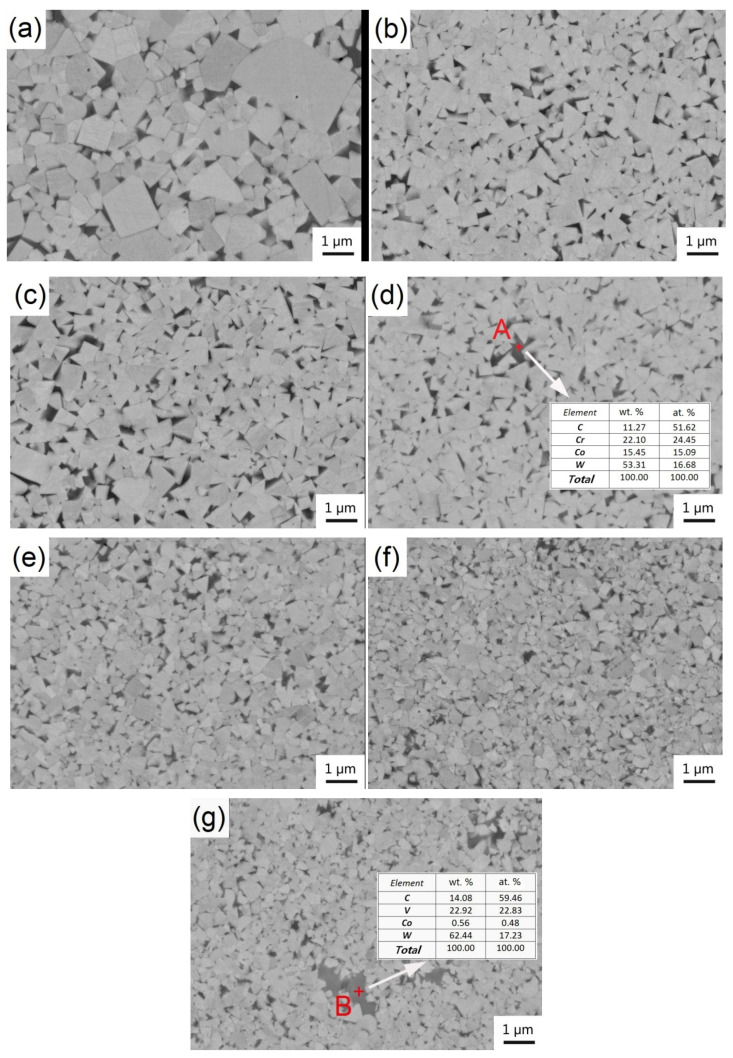
SEM images showing microstructures of WC–6Co–(Cr_2_C_3_/VC) cemented carbides sintered at 1450 °C. (**a**) WC–6Co; (**b**) WC–6Co–0.25Cr_3_C_2_; (**c**) WC–6Co–0.6Cr_3_C_2_; (**d**) WC–6Co–1.0Cr_3_C_2_; (**e**) WC–6Co–0.25VC; (**f**) WC–6Co–0.6VC and (**g**) WC–6Co–1.0VC.

Figure 3 shows the microstructures of sintered WC–6Co–(Cr_2_C_3_/VC) cemented carbides. In the micrograph, bright contrast is WC phase and dark represent Co phase. It can be seen from Figure 3a, there are some coarse WC grains formed in sample 1# due to abnormal grain growth (AGG). With the addition of Cr_2_C_3_/VC, no obvious AGG is observed in the samples 2#–7# and WC grains present obvious refinement. This result implies that Cr_2_C_3_ and VC have significant effect on inhibiting grain growth. Moreover, the VC-added cemented carbide exhibits the finer grains, which indicates the inhibition effect of VC is higher than that of Cr_2_C_3_.

**Figure 4:**
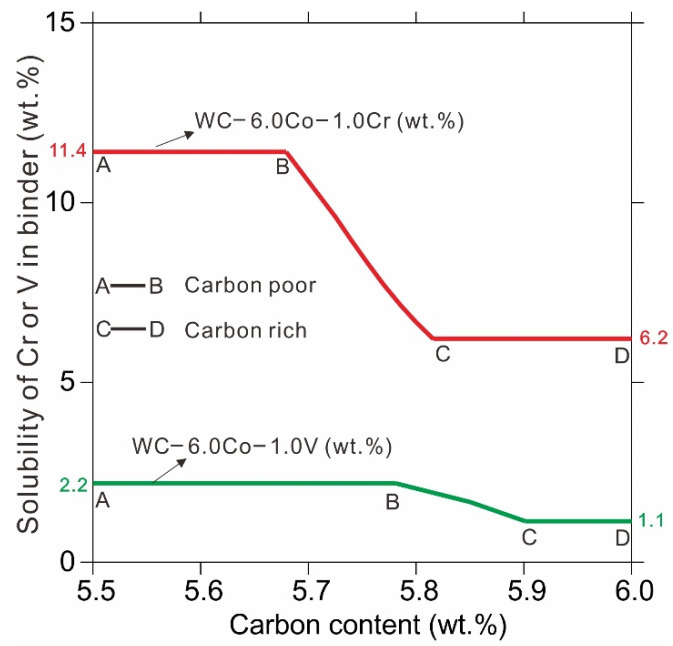
Calculated saturated solubility of Cr and V in Co binder phase based on the thermodynamic database developed by Peng et al. [25].

Figure 4 presents the calculated saturated solubility of Cr and V in Co binder phase based on the thermodynamic database developed by Peng et al. [25]. Due to the fact that the carbon content has a significant influence on binder phase composition, Figure 4 presents the solubility of GGIs in the binder phase along with carbon content. The carbon content ranges of A–B and C–D in Figure 4 represent the existence of carbon-poor phase (η) and carbon-rich phase (graphite), respectively. The carbon content in cemented carbides has to be carefully controlled to a certain range in order to avoid the undesirable carbon-poor (η) and carbon-rich (graphite) phases [25], being in the range of B–C in Figure 4. The calculated solubilities 6.2–11.4 wt. % of the binder phase for Cr_3_C_2_ and 1.1–2.2 wt. % for VC specify the upper and lower values in practical alloys. According to the saturated solubilities of GGIs in the binder phase, the maximum doping amounts of Cr_3_C_2_ and VC in WC-6Co cemented carbides can be deduced, i.e., 0.79 wt. % for Cr_3_C_2_ and 0.16 wt. % for VC. From Figure 3d,g, it is clear that some GGI–based carbides are formed due to the doping Cr_3_C_2_ and VC exceeds their maximum solubilities in the cobalt binder phase. EDS analysis of the precipitates confirmed that the GGI–based carbide in sample 4# is Co-rich Cr-carbide and in sample 7# is Co-deficient V-carbide. In addition, the size of V-carbide is larger than that of Cr-carbide, leading to a more heterogeneous microstructure. Hence, the Co-deficient V-carbides are more detrimental to the mechanical properties.

### 3.2. Effect of Cr_3_C_2_/VC Content on WC Grain Size

**Figure 5:**
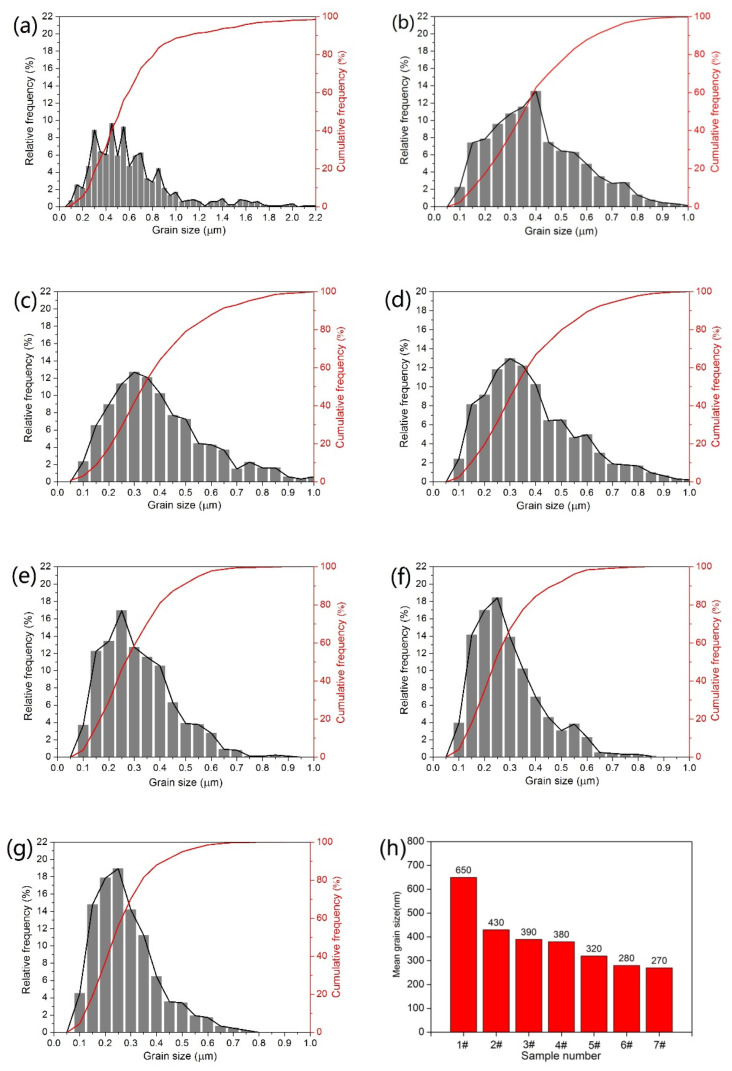
Grain size distribution and average grain size of WC–6Co–(Cr_2_C_3_/VC) cemented carbides. (**a**) WC–6Co; (**b**) WC–6Co–0.25Cr_3_C_2_; (**c**) WC–6Co–0.6Cr_3_C_2_; (**d**) WC–6Co–1.0Cr_3_C_2_; (**e**) WC–6Co–0.25VC; (**f**) WC–6Co–0.6VC; (**g**) WC–6Co–1.0VC and (**h**) mean grain size.

The grain size was measured via linear–intercept method based on SEM images of Figure 3. Figure 5 shows the average size and size distribution of WC grains in WC–6Co– (Cr_3_C_2_/VC) cemented carbides. It is clear that grain size distribution becomes narrow and average grain size reduces significantly with the addition of Cr_3_C_2_/VC. As shown in Figure 5a,g, the size distribution of sample 1# is from 0.1 to 3.0 μm and average size is 0.65 μm. The average grain size first rapidly decreases and then slightly decreases with the increasing Cr_3_C_2_/VC content, as shown in Figure 5h. With the addition of Cr_3_C_2_, the average grain size of samples 2#, 3# and 4# decrease to 0.43, 0.39 and 0.38μm and decrease by 33.8%, 40.0% and 41.5% compared to sample 1#, respectively. From Figure 5a–c, the proportion of 0.05–0.4 μm WC grains of samples 2# and 3# increase obviously as Cr_3_C_2_ content increases from 0 to 0.6 wt. % compared to sample 1#. However, with the increase in Cr_3_C_2_ content from 0.6 to 1.0 wt. %, there is no obvious variation in size distribution and average size of sample 4# compared to sample 3#, as shown in Figure 5d,h. With the addition of VC, the average grain size of samples 5#, 6# and 7# decrease to 0.32, 0.28 and 0.27 μm and decrease by 50.8, 56.9 and 58.5% compared to sample 1#, respectively. This result shows that the inhibition effect of VC is better than that of Cr_3_C_2_. In addition, compared with the samples with Cr_3_C_2_ addition, the samples with VC addition exhibit different size distribution. The proportion of 0.05–0.4 μm WC grains of samples 5#, 6# and 7# are 81.01, 84.51 and 87.92% respectively, which are higher than that of the samples 2#, 3# and 4# (as shown in Table 2). Similarly, as the VC content increases from 0.6 to 1.0 wt. %, the variation in size distribution and average size is slight, as shown in Figure 5f,g. In summary, VC leads to narrower size distribution and smaller grain size compared to Cr_3_C_2_.

**Figure 6:**
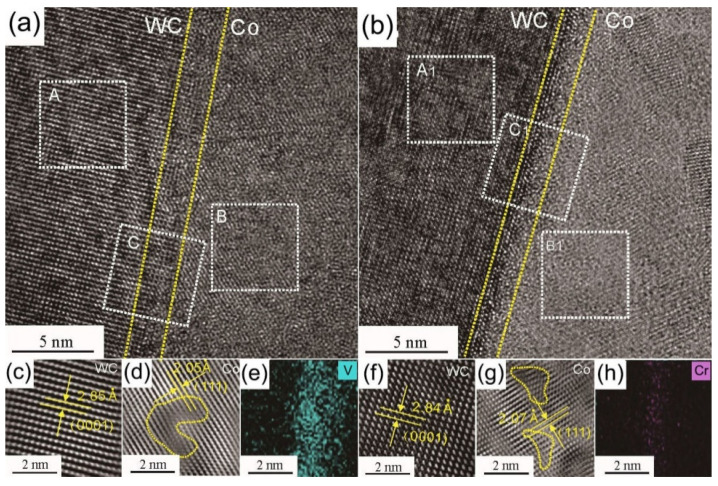
HRTEM images of the WC/Co interface areas doped with V and Cr. (**a**) WC–6Co–0.6VC; (**b**) WC–6Co–0.6Cr_3_C_2_; (**c**) FFT of region A; (**d**) FFT of region B; (**e**) HREDX result of region C; (**f**) FFT of region A1; (**g**) FFT of region B1; (**h**) HREDX result of region C1.

Figure 6a,b show the HRTEM images of the WC/Co interfaces in WC–6Co–0.6VC (Sample 6#) and WC–6Co–0.6Cr_3_C_2_ (Sample 3#), respectively. Figure 6c,d,f,g present the fast Fourier transformation (FFT) of regions A, B, A1 and B1, respectively. It is generally known that the solid solubility of Cr or V in WC is negligible. The (0001) interplanar distances of the WC phase in Samples 3# and 6# are similar. According to Figure 4, the solid solubility of Cr in Co binder phase is larger than that of V. As shown in Figure 6d,g, larger (111) interplanar distance of the Co binder phase doped with Cr can be detected. Figure 6e,h show the elemental mapping of V in region C and Cr in region C1, respectively. It is clear that with the same doping amount of VC and Cr_3_C_2_ (0.6 wt. %), the segregation concentration of V at WC/Co interface is much larger than that of Cr. Consequently, the better inhibition of VC than Cr_3_C_2_ can be attributed to the much easier segregation behavior of V at WC/Co interface compared with Cr.

### 3.3. Effect of Cr_3_C_2_/VC Content on WC Grain Morphology

**Figure 7:**
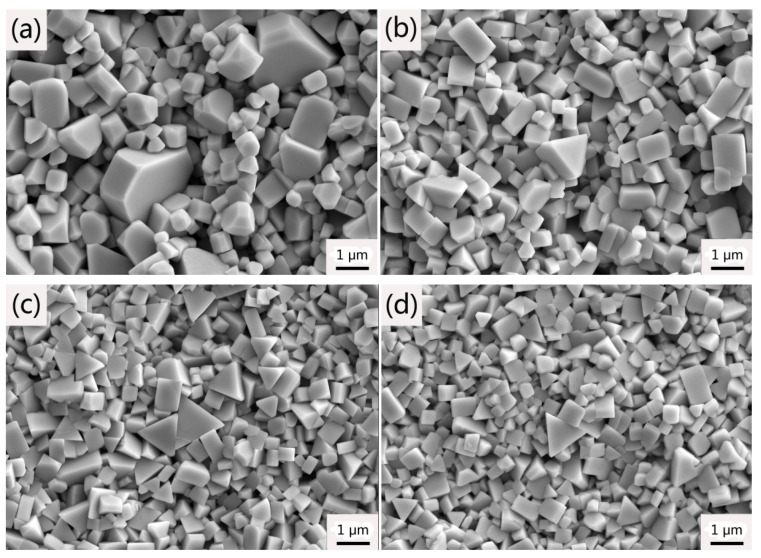

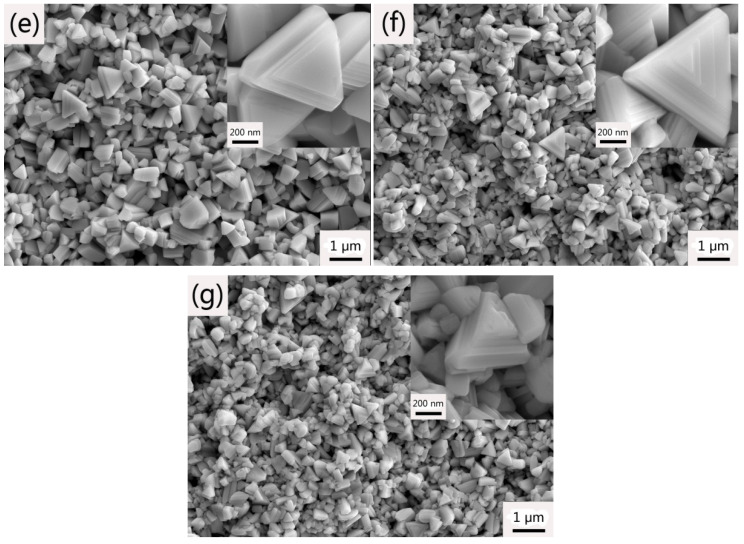
WC grain morphologies of WC–6Co–(Cr_2_C_3_/VC) cemented carbides sintered at 1450 °C. (**a**) 1#; (**b**) 2#; (**c**) 3#; (**d**) 4#; (**e**) 5#; (**f**) 6# and (**g**) 7#.

Figure 7 shows the WC grain shapes of WC–6Co–Cr_3_C_2_/VC cemented carbides sintered at 1450 °C for 1 h. According to Figure 7a, the WC grain in sample 1# without GGI additive exhibits a truncated triangular prism shape, which is the typical equilibrium morphology of WC grains [26]. However, with the addition of Cr_3_C_2_/VC, WC grain shapes transform to triangular prismatic shape and stepped triangular prismatic shape, respectively, as shown in Figure 7b–g. Compared to truncated triangular prism shape grains caused by anisotropic growth, triangular prismatic shape grains and stepped triangular prismatic shape grains are the product of isotropic growth. The anisotropic coarsening of WC grains is mainly controlled via the 2D nucleation and growth mechanism [27,28].

**Figure 8:**
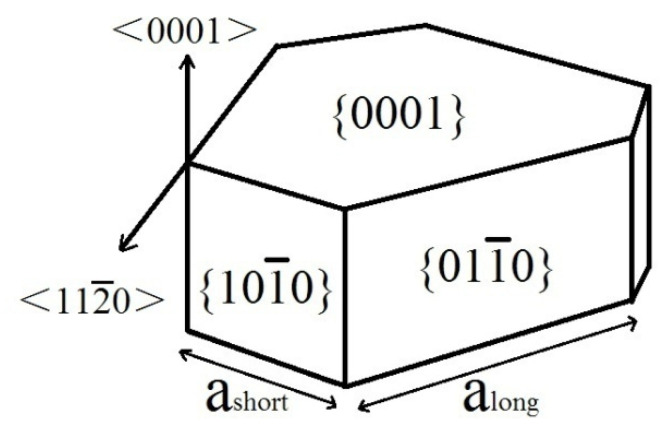
Schema of the WC grain shape in Co binder.

As well known, the WC grains distributed in the Co matrix usually generate two sets of three equivalent prismatic {10$\bar{1}$0} facets rather than six equivalent prismatic {10$\bar{1}$0} facets, and two basal (0001) facets [29]. The set of prismatic {10$\bar{1}$0} facets and prismatic {01$\bar{1}$0} facets are denoted type I and II, respectively. Moreover, the set of prismatic {01$\bar{1}$0} facets is more developed than the set of prismatic {10$\bar{1}$0} facets. Schema of the WC grain shape in Co binder is shown in Figure 8. With the addition of Cr_3_C_2_/VC, the (Cr, W) Cx segregation layers and (V, W) Cx segregation layers formed at (0001) basal facets and {10$\bar{1}$0} prismatic facets of WC grains would reduce surface energies and improve the energy barrier for 2D nucleation and growth [30,31,32]. Therefore, the growth rate of (0001) basal and {10$\bar{1}$0} prismatic facets is inhibited and the {01$\bar{1}$0} prismatic facets grow preferentially [33,34]. As shown in Figure 7b–d, Cr_3_C_2_ addition induces triangular prismatic shape grains. With increasing the Cr_2_C_3_ content, the WC shape becomes sharper and regular as the isotropic growth is enhanced. However, VC addition induces stepped triangular prismatic shape grains. The reason can be attributed to the ratio of (V, W) Cx segregation layers between (0001) basal facets and {10$\bar{1}$0} prismatic facets is much higher than that of (Cr, W) Cx [35,36]. Therefore, WC grains are stacked along [0001] direction to form multi–steps based on (0001) basal planes [37]. As VC content increases from 0.25 to 0.6 wt. %, the WC shape becomes sharper and multi-steps increases significantly due to enhancing isotropic growth. However, the WC shape becomes bulky as the VC content increases to 1.0 wt. %, as shown in Figure 7g. The explanation for this phenomenon could be that the continuous growth of WC grains is strongly inhibited due to the high VC content.

### 3.4. Effect of Cr_3_C_2_/VC Content on the Mechanical Properties

**Figure 9:**
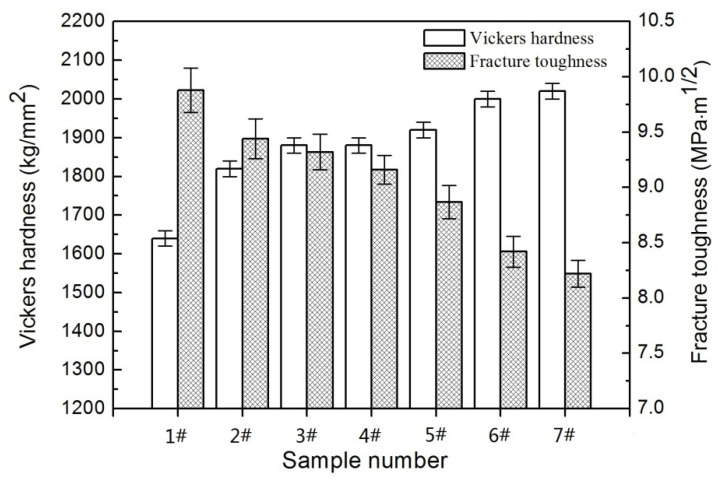
Vickers hardness and fracture toughness of WC–6Co–(Cr_2_C_3_/VC) cemented carbides sintered at 1450 °C. 1#: WC–6Co; 2#: WC–6Co–0.25Cr_3_C_2_; 3#: WC–6Co–0.6Cr_3_C_2_; 4#: WC–6Co–1.0Cr_3_C_2_; 5#: WC–6Co–0.25VC; 6#: WC–6Co–0.6VC; 7#: WC–6Co–1.0VC.

Hardness and fracture toughness are two most important mechanical properties of ultrafine cemented carbides, which should be focused on. Figure 9 shows the Vickers hardness and fracture toughness of WC–6Co cemented carbides with various GGI concentration. It is obvious to see that the hardness initially rapidly increases and then slowly ascends with the rising GGI content. Sample 1# without GGI introduction exhibits the minimum hardness of 1640 kg/mm^2^. With increasing the Cr_2_C_3_ content, the hardness of samples 2#, 3# and 4# are 1820, 1880 and 1890 kg/mm^2^, increased by 11.0, 14.6 and 15.2% compared to sample 1#, respectively. Similarly, with increasing the VC content, the hardness of samples 5#, 6# and 7# are 1920, 2000 and 2020 kg/mm^2^, increased by 17.0, 22.0 and 23.2% compared to the non-added one, respectively. As well known, the hardness of the cemented carbides can be enhanced as the decrease of grain size according to the Hall-Petch relation [38]. The smaller the grain sizes of the samples, the higher the hardness. Therefore, the variation of hardness is consistent with that of grain size. As a result, sample 7# possesses the maximum hardness of 2020 kg/mm^2^ due to the smallest grain size.

**Figure 10:**
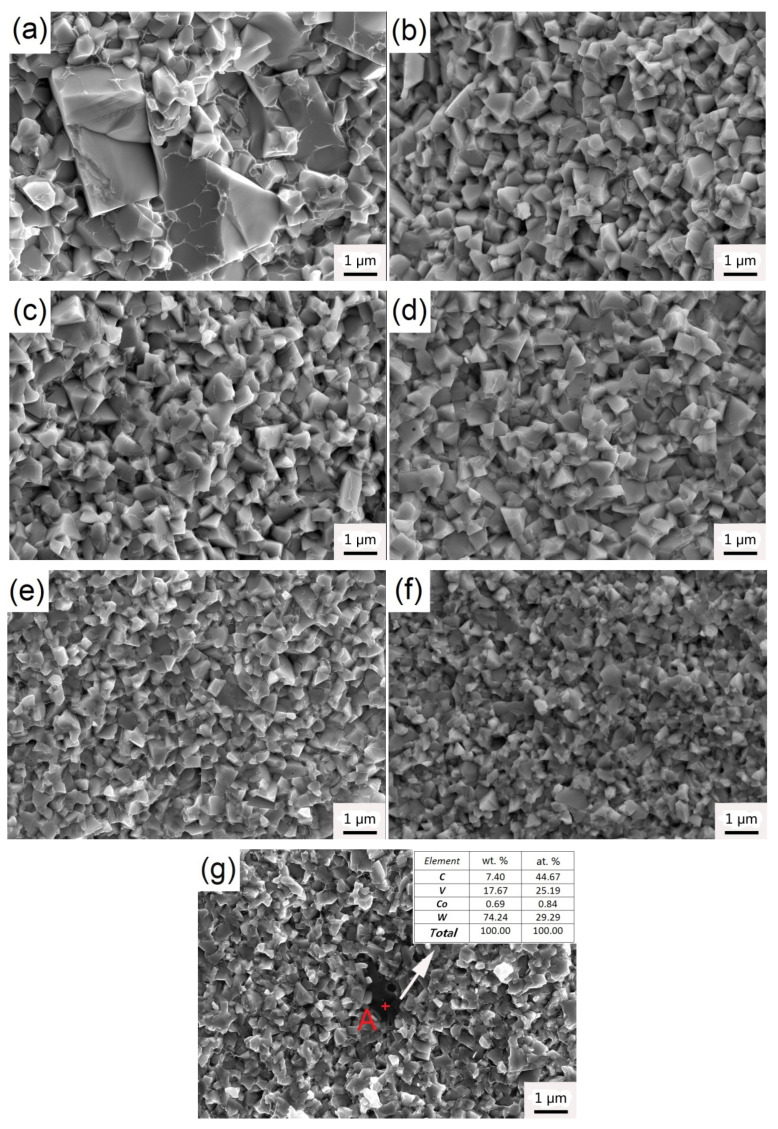
SEM images showing fracture surfaces of WC–6Co–(Cr_2_C_3_/VC) cemented carbides. (**a**) WC–6Co; (**b**) WC–6Co–0.25Cr_3_C_2_; (**c**) WC–6Co–0.6Cr_3_C_2_; (**d**) WC–6Co–1.0Cr_3_C_2_; (**e**) WC–6Co–0.25VC; (**f**) WC–6Co–0.6VC; (**g**) WC–6Co–1.0VC.

However, it was found that the fracture toughness decreases gradually as the Cr_2_C_3_/VC content increases, as shown in Figure 9. As well known, the fracture toughness is inversely proportional to the hardness and thus the fracture toughness decreases as the hardness increases [39]. Among all studied samples, sample 1# has the highest fracture toughness of 9.88 MPa.m^1/2^ due to the lowest hardness. In addition, transgranular fracture occurs frequently due to the existence of micron-sized WC grains, as shown in Figure 10a. Crack passes directly through WC grains and greatly consumes the crack propagation energy, resulting in the increasing of fracture toughness. With the addition of GGI, the grain size decreases significantly and almost no coarse grains exist. According to Figure 10b–g, the transgranular fracture occurs rarely and fracture is mainly carried out along the WC/Co grain boundary (intergranular fracture). As the intergranular strength is lower than intragranular strength, the intergranular fracture cannot effectively prevent crack propagation, resulting in low fracture toughness. Generally, the inhibition efficiency increases with the increasing GGI content. As a result, the fracture toughness decreases as the GGI content increases. In addition, the addition of GGI leads to low WC/Co interface coherency, which makes it easy for cemented carbides to fracture along WC/Co interfaces. Compared to Cr_2_C_3_, VC has higher inhibition efficiency and has stronger effect on reducing WC/Co interface coherency. Hence, the samples with VC addition have lower fracture toughness than the samples with Cr_2_C_3_ addition. Finally, sample 7# with 1.0 wt. % VC has the lowest fracture toughness of 8.22 MPa·m^1/2^.

**Figure 11:**
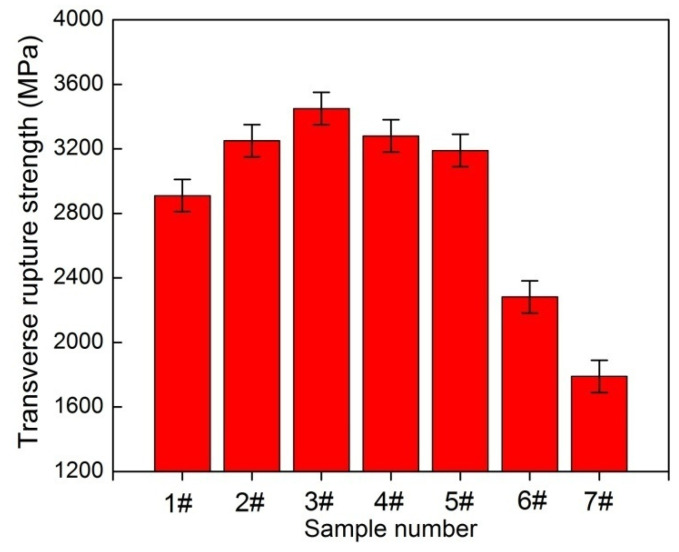
Transverse rupture strength of WC–6Co–(Cr_2_C_3_/VC) cemented carbides sintered at 1450 °C. 1#: WC–6Co; 2#: WC–6Co–0.25Cr_3_C_2_; 3#: WC–6Co–0.6Cr_3_C_2_; 4#: WC–6Co–1.0Cr_3_C_2_; 5#: WC–6Co–0.25VC; 6#: WC–6Co–0.6VC; 7#: WC–6Co–1.0VC.

Figure 11 shows the TRS of samples sintered at 1450 °C for 1 h. With the addition of Cr_2_C_3_, the TRS of samples 2#, 3# and 4# are 3250, 3450 and 3280 MPa and improve by 12, 19 and 13% compared to sample 1# of 2900 MPa, respectively. This result can be attributed to the following two aspects. On the one hand, with the addition of Cr_2_C_3_, the total amount of the interfacial area between the WC grain and the Co phase increases as the grain size decreases, causing a significant enhancement in the TRS. On the other hand, the Co binder phase is strengthened by the addition of Cr_2_C_3_. The TRS achieves the maximum value as 0.6 wt. % Cr_3_C_2_ added and slightly decreases as the Cr_3_C_2_ content increases to 1.0 wt. %. The reason can be attributed to the formation of Co–rich carbides of (Cr,W,Co)C as 1.0 wt. % Cr_3_C_2_ is added. However, the TRS of samples first increases and then sharply decreases (2910→3200→2280→1790 MPa) with the increasing VC content (1#→5#→6#→7#: 0→0.25→0.6→1.0 wt. %). With the addition of 0.25 wt. % VC, the TRS of sample 5# is 3200MPa and increases by 10% compared to sample 1#. At this stage, the grain refinement is the primary reason of the increase in TRS. With the increasing VC content from 0.25 to 1.0 wt. %, the TRS of samples 6# and 7# are 2280 and 1790 MPa and decrease by 21.6% and 32.3% compared to sample 1#, respectively. The sharply decrease in TRS can be attributed to the following three aspects. First, VC has the strongest effect on reducing WC/Co interface coherency compared to other GGI [40]. The higher the VC content, the lower the bonding strength of WC and Co. Second, the stepped triangular prism structure is easy to cause stress concentration and increases the sensitivity of fracture. With the increase of VC content, the number of multi–steps increases significantly. Third, the formation of (V,W)C carbides leads to the heterogeneous microstructure and deteriorates the TRS due to its poor wettability with Co phase. Obviously, change in TRS is a comprehensive outcome of reduction of grain size, low fraction of WC/Co interface coherency and stepped triangular prism grain shape. Therefore, it is evident that GGI and adding amount play a key role in mechanical properties and microstructure. In short, sample 3# with 0.6 wt. % Cr_3_C_2_ addition has good comprehensive mechanical properties compared to other samples, its hardness, fracture toughness and TRS are 1880 kg/mm^2^, 9.32 MPa·m^1/2^ and 3450 MPa, respectively.

## 4. Conclusions

With the increasing Cr_3_C_2_/VC content, the grain size first rapidly decreased and then slightly decreased, and VC led to finer grain size and narrower size distribution due to higher inhibition efficiency. HRTEM/EDS analysis of the WC/Co interface indicates that the segregation concentration of V is much larger than that of Cr, which may be a strong response to the higher inhibition efficiency of VC. The addition of Cr_3_C_2_ induced triangular prism shape WC grains while VC generated stepped triangular prism grains. When the doping of Cr_3_C_2_/VC exceeds the maximum solubility in Co phase, Co-rich Cr-carbides and Co-deficient V-carbides would form respectively, which were detrimental to the TRS and impact toughness. The hardness increased with the increasing Cr_3_C_2_/VC content, while the fracture toughness decreased with the increasing Cr_3_C_2_/VC content. With the increasing VC content, the reduction of grain size, stepped triangular prism shape grains and low fraction of WC/Co interface coherency worked together to cause the TRS to first increase and then decrease sharply. The sample with 0.6 wt. % Cr_3_C_2_ addition had good comprehensive mechanical properties, its hardness, fracture toughness and TRS were 1880 kg/mm^2^, 9.32 MPa·m^1/2^ and 3450 MPa, respectively. In this work, a hybrid approach of thermodynamic calculation and experiment has been developed to systematically investigate the doping amount of GGIs on the microstructure and mechanical properties of cemented carbides, which provides an efficient approach for designing the optimal composition during the development of other materials. Besides, the presently obtained microstructure evolutions can be further subjected to phase-field simulations or finite element method for quantitatively controlling microstructure and mechanical properties.

## Figures and Tables

**Table 1:** Composition ratios and number of the WC–6Co–(Cr_2_C_3_/VC) cemented carbides (wt. %).

Cemented Carbides	Number	WC–6Co	Cr_3_C_2_	VC
WC–6Co	1#	100	0	0
WC–6Co–0.25Cr_3_C_2_	2#	99.75	0.25	0
WC–6Co–0.6Cr_3_C_2_	3#	99.4	0.6	0
WC–6Co–1.0Cr_3_C_2_	4#	99.0	1.0	0
WC–6Co–0.25VC	5#	99.75	0	0.25
WC–6Co–0.6VC	6#	99.4	0	0.6
WC–6Co–1.0VC	7#	99.0	0	1.0

**Table 2:** Proportion and total amount of 0.05–0.4 μm grains in samples 1#–7# (%).

Sample	0.05 µm	0.10 µm	0.15 µm	0.20 µm	0.25 µm	0.30 µm	0.35 µm	0.40 µm	Total
1#	0	0.72	2.52	2.16	4.68	8.89	6.37	6.00	31.37
2#	0	2.24	7.40	7.84	9.54	10.74	11.54	13.34	62.66
3#	0	2.34	6.51	8.96	11.33	12.69	12.07	10.22	64.14
4#	0	2.42	8.14	9.14	11.80	12.98	12.20	10.22	66.91
5#	0	3.66	12.23	13.40	16.93	12.71	11.54	10.52	81.01
6#	0	3.95	14.13	16.98	18.40	13.86	10.23	6.94	84.51
7#	0	4.51	14.78	17.85	18.92	14.17	11.22	6.46	87.92

## Data Availability

Data is contained within the article.
